# Trends in acute specialist contacts following a primary care contact 2012–21—a registry-based study

**DOI:** 10.1080/02813432.2026.2677785

**Published:** 2026-06-02

**Authors:** Målfrid A. Nummedal, Andreas Asheim, Johan Håkon Bjørngaard, Kjartan Sarheim Anthun, Lars Petter Bjørnsen, Ellen Rabben Svedahl

**Affiliations:** ^a^ Trondheim Emergency Department Research Group (TEDRG), Department of Circulation and Medical Imaging, Faculty of Medicine and Health Sciences, Norwegian University of Science and Technology (NTNU), Trondheim, Norway; ^b^ Center for Healthcare Improvement, St. Olav’s Hospital – Trondheim University Hospital, Trondheim, Norway; ^c^ Department of Mathematical Sciences, Norwegian University of Science and Technology (NTNU), Trondheim, Norway; ^d^ Department of Public Health and Nursing, Faculty of Medicine and Health Sciences, Norwegian University of Science and Technology (NTNU), Trondheim, Norway; ^e^ Department of Health Research, SINTEF Digital, Trondheim, Norway; ^f^ Clinic of Emergency Medicine and Prehospital Care, St. Olav’s Hospital – Trondheim University Hospital, Trondheim, Norway

**Keywords:** Acute referral, acute contacts, gatekeeping, primary care, ED crowding

## Abstract

**Background:**

Acute patients in Norway can access specialist services through various pathways, with primary care services having a gatekeeping role. Although national data show declining acute specialist contacts, some emergency departments report increasing visits. This study examined national trends in acute specialist contacts following primary care visits.

**Methods:**

This registry-based cohort study included all Norwegian citizens from 2012 to 2021. We obtained information on patients’ contact with primary care (general practitioner (GP) service and out-of-hours service) before an acute specialist contact. Acute specialist contacts occuring within 10 h of a primary care contact were considered referrals. Incidence rates were estimated using Poisson regression.

**Results:**

Among 12.2 million acute specialist contacts, 21.8% had a preceding out-of-hours service contact, 19.2% had a preceding GP service contact, and 58.9% had no prior primary care contact. From 2012 to 2021, the incidence rate of acute specialist contacts following a primary care contact increased by 18.5% (95% CI: 17.9–19.1), from 91.8 to 109.3 per 1,000 person-years. This trend was observed across all demographic groups, and for both admissions and outpatient visits. The increase was most pronounced for referrals following the out-of-hours service (46.6 to 59.3 per 1,000 person-years), while GP service referrals remained stable.

**Conclusions:**

Acute specialist contacts declined over time, while the proportion and incidence rate of those following a primary care contact increased, particularly following out-of-hours services. The consistent trend across demographic groups suggests that changes in healthcare organisation and service use, rather than population changes, are contributing to the observed trend.

## 1. Introduction

Acute patients access Norwegian hospitals through various pathways and comprise more than two-thirds of all hospital admissions nationwide [1]. As acute patients put strain on the health services, Norwegian national initiatives have been implemented with the aim of reducing acute hospitalisations through more efficient service delivery and improved communication between primary care and specialised healthcare [2–4]. In 2012, the Norwegian Coordination Reform [4] was implemented to better coordinate primary care and specialised healthcare [5].

In Norway, patients typically first contact their regular general practitioner (GP) or municipal out-of-hours services to access specialised healthcare [5], or are transported by emergency medical services. Data from 2014 show that GPs and out-of-hours services initiated almost 2/3 of all acute hospital admissions [6]. While research indicates that improved access to primary care reduces avoidable hospitalisations [7–9], the increasing demands on primary care services [10,11] could potentially compromise the effectiveness of the gatekeeper function if physicians face patient volumes that exceed their capacity to deliver appropriate care. Previous studies of referrals have focused on the primary care perspective [12–19], examining patient and physician characteristics rather than hospital utilisation patterns. Some studies have examined primary care contacts before acute hospital admissions for a short period of time [12] or specific patient groups [20]. However, no comprehensive national analysis has examined how primary care referral patterns influence acute hospital utilisation trends over time.

Patients requiring acute specialised healthcare at a hospital are usually routed through the emergency department, resulting in either an admission or an acute outpatient contact. However, direct admission to a ward or a visit to a designated outpatient clinic is also possible, depending on the patient’s medical condition and local organisations. Although national data show a general decline in acute hospital admissions (2013–2017) [2], two Norwegian emergency departments report increased visits in all age groups (2012–2021) [21]. Globally, emergency department crowding is a growing concern, with visits increasing among both older patients with complex conditions [22] and the younger population [23,24]. This apparent contradiction between declining acute hospital admissions nationally and increasing emergency department visits in some regions suggests a broader shift in healthcare utilisation patterns. These divergent trends underscore the need to investigate how primary care services, as traditional gatekeepers to hospital care, relate to these usage patterns.

Using comprehensive registry data from 2012 to 2021, we aim to describe national trends in acute specialist contacts following primary care visits (GP service and out-of-hours service), and to examine the extent of primary care as a prehospital pathway to acute specialist care.

## 2. Methods

This is a cohort study using registry data from all Norwegian citizens between January 1st, 2012 and December 31st, 2021.

### 2.1. Study setting

The Norwegian healthcare system operates on two levels: primary healthcare and specialised healthcare. Municipalities manage primary healthcare, including regular GP services and out-of-hours services, which are the focus of this study. Regional health authorities manage specialised healthcare [5]. In this study, an acute patient refers to an individual who presents with a sudden onset of symptoms or illness that may require prompt medical attention. This includes both potentially emergency and urgent conditions.

The foundation of primary healthcare rests on the regular GP service, with over 99% of citizens registered with a named GP in 2021 [25]. Regular GPs serve the population during office hours (mainly from 8 am to 4 p.m. on weekdays). Outside these hours, out-of-hours medical care is organised according to municipality size [26]. Smaller municipalities may have GPs or other physicians on call, while urban areas operate designated urgent care wards [27] open both daytime and after-hours. Access to specialised healthcare in Norway follows a structured referral system, with self-referral not normally permitted. In general, citizens require a referral from either their GP or an out-of-hours physician, except when transported by emergency medical services following an emergency call or women in labour. In addition, patients with minor injuries may access specialist-run urgent care centres (trauma clinics) in the two largest cities, Oslo and Bergen, which can be accessed directly without referral.

### 2.2. Study population

The study population comprised all citizens of Norway from January 1, 2012, to December 31, 2021. We included individuals on January 1, each year, and excluded them on the day of their emigration or death.

### 2.3. Data sources

Norwegian citizens are assigned a unique personal identity number, which enables the linkage between Norwegian registers. For each citizen, data were available on all acute somatic specialist contacts through the National Patient Registry (NPR), and all contacts with GP services and out-of-hours services through the Control and Payment of Health Reimbursement Register (KUHR). Since the registers do not specify referral sources, we made the assumption that acute contacts in specialised healthcare that followed less than 10 h after a GP service or out-of-hours service contact represent referrals from these instances.

#### 2.3.1. The control and payment of health reimbursement register (KUHR) [28]

Primary care physicians, both daytime and after-hours, submit unique claims for reimbursement for each patient contact to the Norwegian Health Economics Administration. These claims are registered in KUHR and include information on the setting, patient, type of contact, diagnosis, and time and date of the contact. We first distinguished between the setting submitting the claim (GP service or out-of-hours service), followed by specific reimbursement codes to categorise contact types (excluding non-relevant services such as laboratory tests and electronic correspondence). See Supplementary File 1 for details on the selected codes. We obtained the number and type of contacts both for GP services and out-of-hours services for each citizen, each year. We categorised contacts based on the service providing the contact, not the individual physician’s primary role (the attending physician can function as a GP during regular hours and work in out-of-hours services in the evening). Contacts with nursing home physicians (including out-of-hours physicians’ contacts in nursing homes) are not registered in this system and, therefore, are not included in this study.

#### 2.3.2. The Norwegian patient registry (NPR) [29]

**Figure 1.**
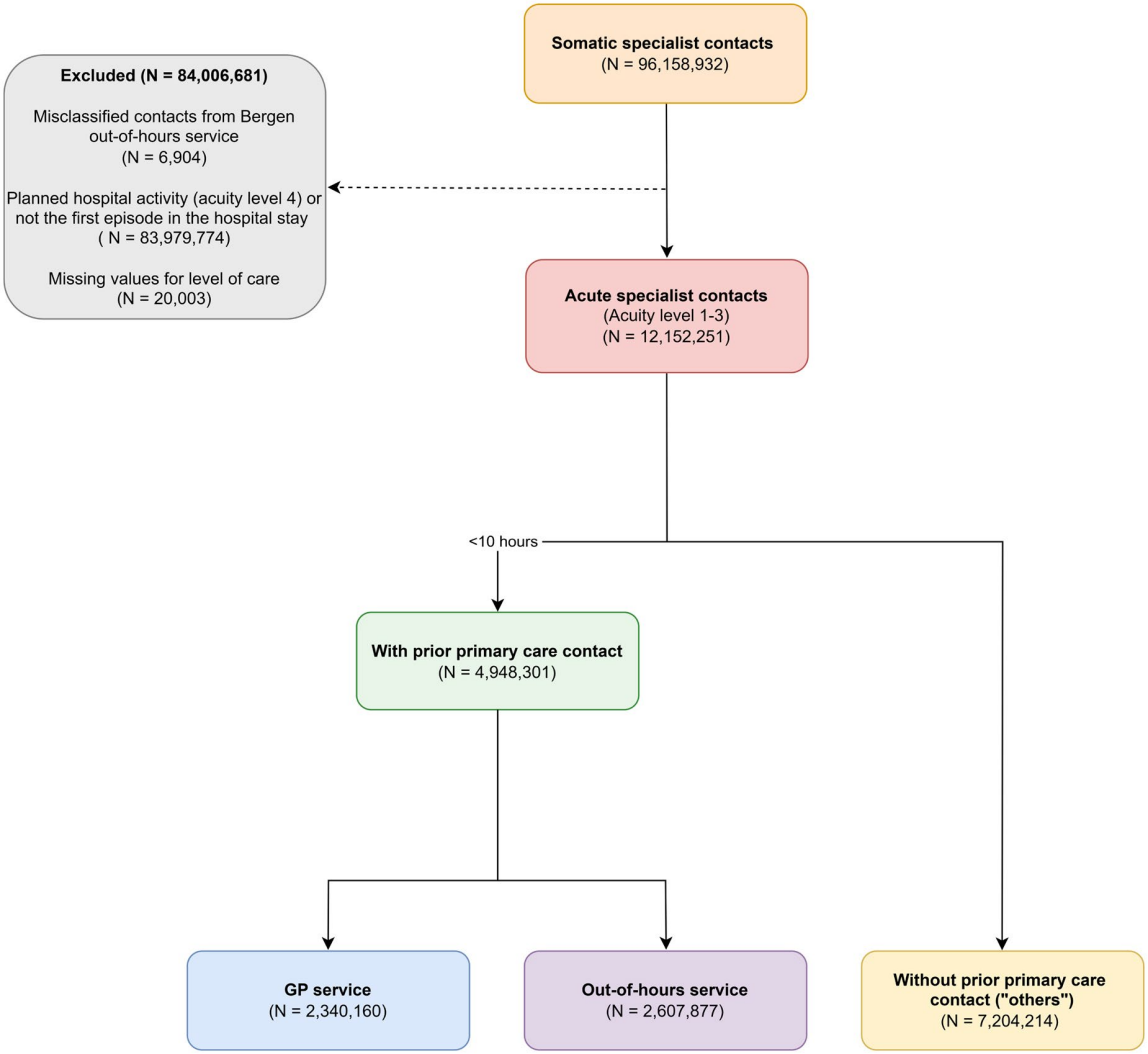
Flowchart of data cleaning methods, 2012–2021. Highlighting prehospital pathways through GP services and out-of-hours services. Other access routes are grouped under ‘others’. GP = General practitioner. Others = Transported with emergency medical services, referral from nursing homes, other institutional physicians referrals, private practice referrals, women in labour, contacts with the trauma clinics in Oslo and Bergen, and internal referrals within the hospital

The Norwegian Directorate of Health maintains the Norwegian Patient Registry, which records all specialised healthcare contacts with hospitals, outpatient clinics, or contracted specialists who provide outpatient care through individual agreements with regional health authorities. This includes emergency department visits, although these contacts cannot be identified as a separate category in the registry. For this study, we included admissions, outpatient visits and daycare treatment (levels of care). We obtained patient information and time of contact. In NPR, contacts are categorised by acuity (levels 1–3 unplanned, 4 elective), elective contacts were excluded. Because transfers between units during a single hospital stay generate additional episodes in NPR, we included only the first episode of each stay to ensure we only counted one contact per patient visit. Misclassified records from Bergen out-of-hours service (2013–2015) were also identified and excluded. An overview of the data cleaning methods is shown in Figure 1.

##### 2.3.2.1. Other data sources

Statistics Norway provided demographic information, and the date of death was obtained from the Norwegian Cause of Death Registry.

### 2.4. Variables

We identified three main types of contacts: primary care contacts, acute specialist contacts, and acute specialist contacts following a primary care contact. The dataset contained information on the total number of each contact for every person, every year.

1. ‘Primary care contacts’ included registered contacts in KUHR from GP services or out-of-hours services, categorised by reimbursement codes as office visits, home visits, online contacts, or telephone contacts (Supplementary file 1).
2. ‘Acute specialist contacts’ included acute specialised healthcare contacts recorded in NPR (Figure 1). Contacts were categorised as either admissions or outpatient contacts, combining daycare treatment and outpatient visits from NPR.
3. ‘Acute specialist contact following a primary care contact’ included acute specialist contacts with a previous primary care contact within 10 hours, representing likely referrals from primary care. This 10-hour time frame was considered a reasonable interval to account for geographic variations in travel times to hospitals in Norway. Because GPs and out-of-hours physicians may not always file claims immediately during emergency contacts, we also included delayed compensation claims filed within 8 hours of the specialist contact. When multiple prior contacts existed, the most recent contact was prioritised, with prehospital claims prioritised over delayed submissions.

If no prior primary care contact was found in KUHR, the specialist contact was categorised as ‘others’, representing all pathways not captured in the KUHR database. This category primarily represents patients arriving with emergency medical services, nursing home referrals (also ambulatory out-of-hours physicians referring from nursing homes), other institutional physicians’ referrals, specialist referrals (within or between hospitals, or from contract specialists), women in labour, and contacts at the trauma clinics in Oslo and Bergen. Some contacts in this category may also represent return visits due to disease deterioration following an earlier specialist contact.

Patient demographics included gender and age (calculated as the contact year minus birth year), categorised as: 0–17 years, 18–66 years, 67–79 years, and 80 years or older [2]. We also obtained persons’ days alive each year (person-years at risk).

### 2.5. Statistical methods

We computed annual counts of primary care contacts, by GP service and out-of-hours service, as well as acute specialist contacts, with and without a prior primary care contact. We used Poisson regression models with robust variance estimation to model rates of contacts, adjusting for calendar year as a categorical variable, and using log person-years at risk as an offset parameter to account for population changes. In additional analyses, we included age and gender as categorical variables, with separate analyses of unique patients and total contacts to examine whether frequent healthcare users generate multiple contacts, as individuals may access healthcare services multiple times per year [30].

For each analysis, we reported estimated incidence rates per 1,000 person-years with 95% confidence intervals (CIs). Additionally, we summarised the types of primary care contacts (office visits, home visits, online contacts, and telephone contacts) preceding acute specialist contacts, presenting these as total yearly counts. Demographic characteristics were described using standard descriptive statistics. All analyses were conducted using STATA version 18.

#### 2.5.1. Sensitivity analysis

Since the trauma clinics in Norway’s two largest cities, Oslo and Bergen, differ from the service organisation in the rest of Norway, we performed sensitivity analyses excluding contacts from these services. As they cannot be identified separately in the registry data but are reported together with Oslo University Hospital HF and Helse Bergen HF, we excluded these two hospital trusts in the sensitivity analyses. All models were then re-estimated on this restricted dataset.

### 2.6. Ethics

This project was approved by the regional ethics committee (2016/2158/REK midt) and later expansions. Informed consent was not deemed necessary.

## 3. Results

From 2012 to 2021, we identified 96.2 million somatic specialist contacts in Norway. After exclusion of planned activity, non-first episodes, records with missing level of care, and misclassified out-of-hours service contacts (Figure 1), a total of 12.2 million acute specialist contacts remained for analysis. Among these, 1.0 million (8.2%) were contacts with contract specialists outside hospitals, and 4.95 million had a prior registered primary care contact.

During the study period, the number of primary care contacts increased from 21.5 million to 24.8 million per year, while acute specialist contacts decreased from 1.6 million to 1.0 million per year. The Norwegian population increased from 5.0 million to 5.3 million in the same period (Table 1).

**Table 1.** Primary care contacts, acute specialist contacts in total and following a primary care contact, and unique citizens, Norway 2012–2021 (in millions).

	Primary care contacts *N* = 229.4.		Acute specialist contacts *N* = 12.2.			
	GP [1] service *N* = 207.16.	OOH [2] service *N* = 22.21.	Following GP contact [3] *N* = 2.34.	Following OOH contact [3] *N* = 2.61.	Total [4] *N* = 12.2.	Unique citizens *N* = 5.9.
Age (IQR)	43 (24–61)	35 (18–57)	53 (29–72)	49 (25–72)	46 (25–68)	40 (21–58)
Women (%)	53.7	52.5	52.1	50.2	53.2	49.7
**Year**						
2012	19.8	1.8	0.23	0.24	1.6	5.0
2013	20.0	1.8	0.23	0.24	1.6	5.0
2014	20.6	1.8	0.24	0.24	1.4	5.1
2015	21.1	1.8	0.24	0.25	1.2	5.2
2016	21.6	1.9	0.23	0.26	1.1	5.2
2017	21.4	1.9	0.24	0.26	1.1	5.3
2018	20.5	2.0	0.24	0.28	1.1	5.3
2019	20.2	2.1	0.24	0.29	1.1	5.3
2020	20.9	3.4	0.22	0.27	1.0	5.4
2021	21.2	3.7	0.23	0.28	1.0	5.3

^1^ General practitioner.

^2^ Out-of-hours.

^3^ The primary care contact was registered within 10 h prior to the acute specialist contact.

^4^ Represents acute specialist contacts both with and without a prior primary care contact.

### 3.1. Acute specialist contacts following a primary care contact

**Figure 2.**
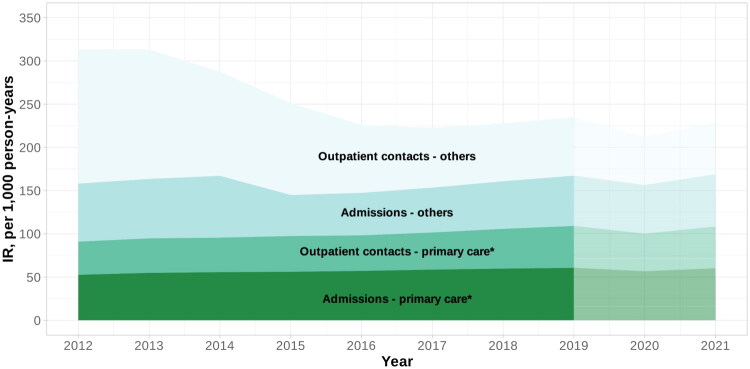
Estimated incidence rate per 1,000 person-years of acute specialist contacts, following a contact with primary care* (within the previous 10 h) or without (others), by type of specialist contact (admission or outpatient contact), 2012–2021. The last two years indicates the period with the COVID-19 pandemic. *General practitioner service or the out-of-hours service

Between 2012 and 2021, the incidence rate of acute specialist contacts following a primary care contact increased by 18.5% (95% CI: 17.9–19.1%), from 91.8 (95% CI: 91.5–92.1) to 109.3 (95% CI: 108.8–109.7) per 1,000 person-years (Supplementary file 2). Overall, 41.1% (95% CI: 35.6–46.6) of acute specialist contacts occurred after a primary care contact. When the acute specialist contacts following a prior primary care contact were categorised as admissions or outpatient contacts, admissions were consistently higher in proportion throughout the study period, although outpatient contacts without a prior primary care contact (‘others’) were the largest category overall (Figure 2).

**Figure 3.**
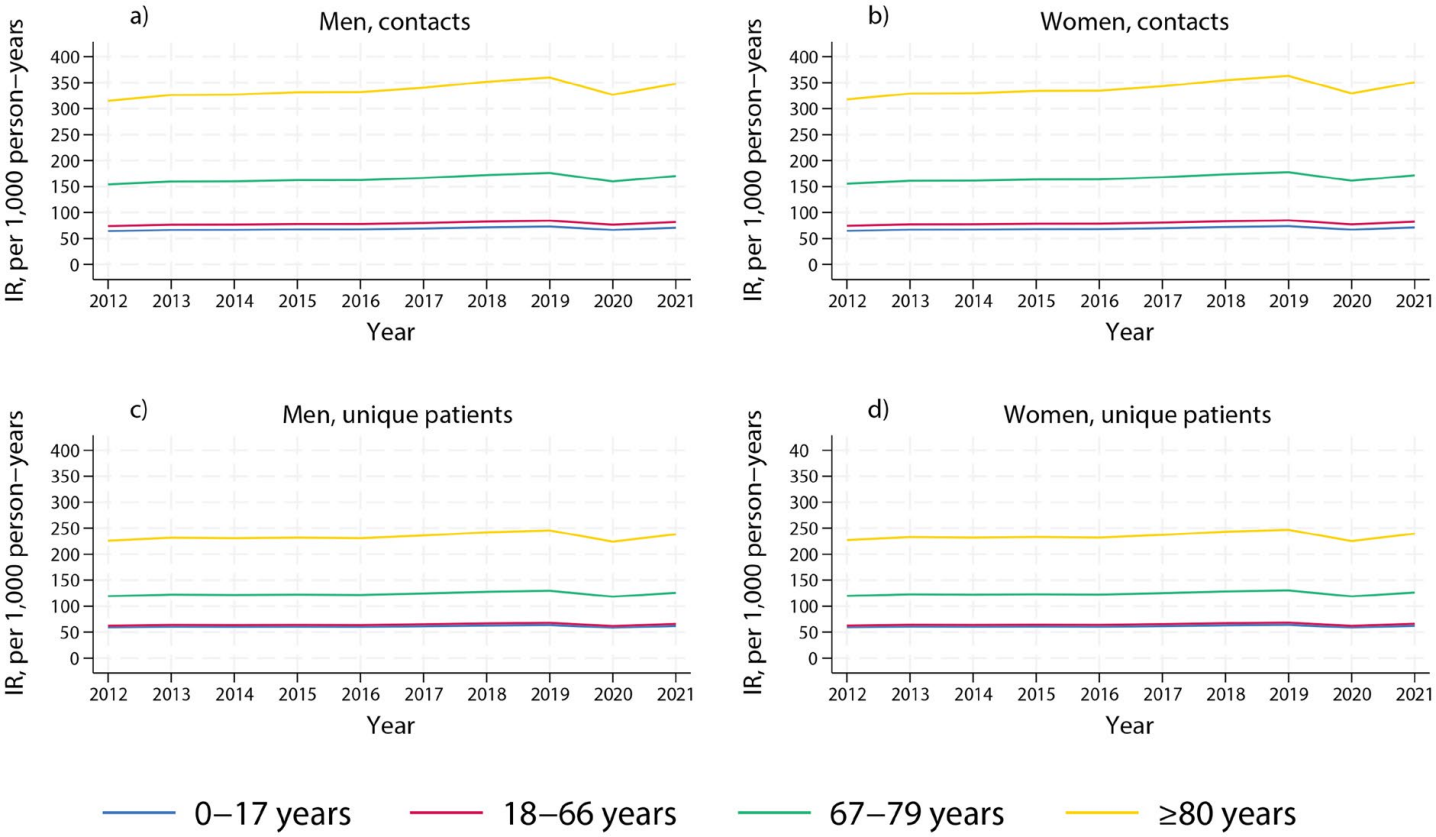
Estimated incidence rate per 1,000 person-years of acute specialist contacts following a contact with general practitioner service or out-of-hours service (within the previous 10 h), by age, showing total contacts (a: men, b: women), and unique patients (c: men, d: women), 2012–2021.

The estimated incidence rates of acute specialist contacts following a primary care contact increased across all age groups for both men and women during the study period (Figure 3). Women consistently had higher incidence rates than men, with the highest rates observed in patients aged 80 and older. When examining unique patients instead of total acute specialist contacts, the rising trends were similar, although less pronounced.

### 3.2. Type of primary care contact prior to patients’ acute specialist contacts

Between 2012 and 2019, the incidence rate of acute specialist contacts following a GP service contact slightly increased from 45.1 (95% CI: 44.9–45.3) to 48.0 (95% CI: 47.9–48.3) per 1,000 person-years, while contacts following out-of-hours services increased from 46.6 (95% CI: 46.4–46.8) to 59.3 (95% CI: 59.0–59.6) per 1,000 person-years (Supplementary file 3). A temporary dip was observed in 2020 for contacts following both GP and out-of-hours services, with rates returning to approximately pre-pandemic levels (2019) in 2021.

Acute specialist contacts without prior primary care contact (‘others’) declined steadily from 221.1 (95% CI: 221.0–223.2) per 1,000 person-years in 2012 to 117.5 (95% CI: 117.0–118.0) in 2017, remained relatively stable through 2019, then decreased further in 2020, ending at 112.9 (95% CI: 112.4–113.5) in 2021.

**Figure 4.**
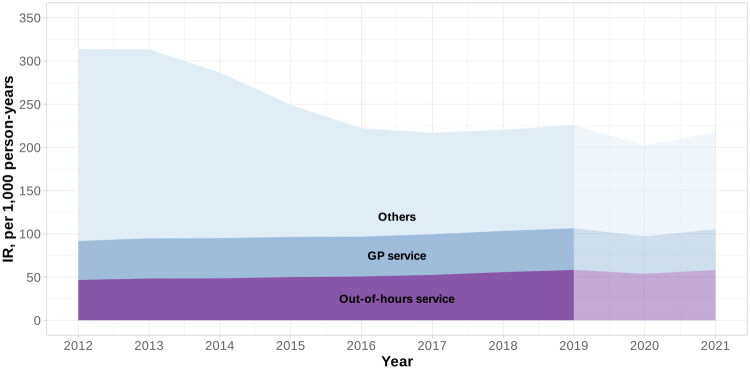
Estimated incidence rates per 1,000 person-years of acute specialist contacts following a contact with either the out-of-hours service, GP (general practitioner) service, or neither (others*) within 10 h before the specialist contact, 2012–2021. The last two years indicates the period with the COVID-19 pandemic. *Acute specialist contacts without a documented primary care contact within the preceding 10 hours.

Across the full study period, 19.2% (95% CI: 17.1–21.4) of acute specialist contacts followed a GP service contact, 21.8% (95% CI: 18.5–25.2) followed an out-of-hours service contact, and 58.9% (95% CI: 53.4–64.4) had no prior registered primary care contact (‘others’) (Figure 4).

Table 2 shows the types of primary care contacts preceding acute specialist contact, in total numbers. Office visits were the most frequent type of contact for both GP services (87.2%) and out-of-hours services (84.2%). For GP services, the number of office visits increased, except for a temporary dip in 2020. Home visits remained relatively stable throughout the study period, while online contacts increased, and telephone contacts decreased. For out-of-hours services, all contact types increased in total numbers, with the largest absolute increase in office visits.

**Table 2.** Total numbers per year of acute specialist contacts with prior primary care contact [1], by type of preceding contact.

	General pracitioner service				Out-of-hours service			
Year	Office (*N* = 2,039,850, 87.2%)	Telephone (*N* = 181,297, 7.7%)	Home visit (*N* = 86,120, 3.7%)	Online [2] (*N* = 32,893, 1.4%)	Office (*N* = 2,195,556, 84.2%)	Telephone (*N* = 223,088, 8.5%)	Home visit (*N* = 186,581, 7.1%)	Online [2] (*N* = 2,652, 0.1%)
2012	197.449	21.981	9.324	0	203.243	15,794	16,870	0
2013	204.123	22.301	8.988	13	209.071	18,659	16.522	0
2014	205.09	21.969	8.671	29	210.443	18,400	16.053	0
2015	205.734	21.633	8.211	44	217.857	17.509	15.938	0
2016	205.118	20.758	8.192	96	220.762	17.424	16,420	0
2017	211.863	17.559	8.104	166	227.819	17.186	17.153	0
2018	214.893	16,190	8.254	466	233,530	24.238	19.349	0
2019	217.332	14.911	8.476	844	239.628	26.982	21.791	0
2020	183.078	12.461	9.144	13.628	209.889	32.043	22.654	1.413
2021	195,170	11.534	8.756	17.607	223.314	34.853	23,840	1.239

^1^ The primary care contact was registered within 10 h prior to the acute hospital contact.

^2^ The reimbursement code for online consultations were in use from 2013.

### 3.3. Sensitivity analyses

For sensitivity analyses, we excluded all contacts from Helse-Bergen HF and Oslo University Hospital HF from acute specialist contacts (*N* = 3,036,396). The analyses produced similar trends for all outcomes but revealed a less pronounced decline in acute specialist contacts without referral from primary care (‘others’) from 2012–2015. See Supplementary files 2 and 3 for the complete numbers on incidence rates.

## 4. Discussion

Between 2012 and 2021, registry-based data showed a 19% increase in the incidence rate of acute specialist contacts recorded within 10 h after a primary care contact in Norway. During the same period, the total number of acute specialist contacts declined, indicating that a higher share of acute patients entered specialist services *via* primary care. By 2021, 48% of all acute specialist contacts had a prior primary care contact with the GP service or the out-of-hours service, compared to 29% in 2012—­representing more than 50,000 additional acute cases assessed by a primary care physician prior to acute specialist care.

The observed increase in assumed primary care referrals was mainly driven by the out-of-hours service, which experienced more than a doubling of patient contacts over the 10-year period, with an increasing number of these contacts resulting in acute specialist referrals. A substantial part of the increase in out-of-hours contacts from 2020 to 2021 is likely attributable to the COVID-19 pandemic. During this time, out-of-hours services in Norway were reorganised to function as testing centres, airway clinics, and telephone helplines as part of the municipal pandemic response, leading to a temporary rise in contact rates [31]. Recent national statistics indicate that activity levels in out-of-hours services have since returned to approximately pre-pandemic levels [32], suggesting that the observed increase does not fully reflect a sustained long-term trend in service utilisation.

Although GP services handled more than 18 million additional annual contacts, out-of-hours services had the highest rate of acute referrals. This pattern likely reflects the fact that out-of-hours services typically handle more urgent cases and may refer patients more quickly due to limited diagnostic capabilities, unfamiliarity with patients, and time pressure. A previous study showed that contacts with out-of-hours services at night and through home visits are more likely to result in an acute referral to specialist care [18]. The larger increase in out-of-hours referrals may also reflect increased patient demand for immediate specialist access after hours, or challenges in daytime GP availability that drive patients to after-hours services for urgent conditions. While the majority of acute referrals resulted in hospital admissions, referrals resulting in outpatient visits increased slightly more.

Increasing referral rates from primary care are also observed in other countries. In the UK, GP-referred specialist visits increased between 2007 and 2015 [33]. In our study, analyses of unique patients compared with total specialist referrals showed similar upward trends, although the increase was less pronounced when examining unique patients. This suggests a possible shift toward more frequent attenders, who would contribute proportionally more referrals over time, although the differences were small. Several other factors may also explain the increasing referral patterns. The increasing complexity of patient care means that more conditions require specialist evaluation [34,35]. The trend may also reflect improved access to specialised healthcare, ensuring more patients receive appropriate diagnostic evaluation and treatment. Notably, while more patients now see specialists after primary care visits, the total number of acute specialist contacts has declined. This may suggest that primary care physicians are treating more patients directly while referring complex cases appropriately.

Our findings align with national data [2] showing an overall reduction in acute specialist contacts. The initial reduction in acute specialist contacts until 2015 coincided with the implementation of Norway’s Coordination Reform. This reform created municipal 24-hour acute care facilities for patients requiring urgent care without hospital services [4]. The reform aimed to reduce acute hospital admissions, particularly for patients over 80 years, by providing appropriate care closer to home, with demonstrated effectiveness in some municipalities [36]. However, the reform primarily targeted patients assessed within primary care and therefore subject to gatekeeping. Not all acute specialist contacts follow this pathway, as some patients are admitted directly to specialist services and are reflected in the category ‘others’. This should be considered when interpreting the observed gatekeeping trends, particularly during the first study years. Unfortunately, data are not available for patients referred to these services during the study period, which would have been helpful to evaluate their full impact.

The overall decline in acute specialist contacts in our data was mainly driven by a substantial reduction in outpatient contacts without a primary care referral (‘others’), while the number of primary care referrals increased. Given the magnitude and rapid timing of this reduction, it is unlikely to reflect true shifts in patient behaviour or healthcare utilisation. Instead, it more plausibly reflects changes in how contacts were registered—such as adjustments to coding practices, reporting routines, or classification rules—although we have not identified such changes. Nonetheless, unrecorded modifications in registry practice could meaningfully influence observed trends, even when the underlying clinical activity remains stable. Hence, contacts for the more recent years 2017–2021 may be more reliable than estimates for the full period.

In addition to potential registry-related shifts, structural differences in healthcare organisation may also contribute to the observed patterns. In Norway’s two largest cities, Oslo and Bergen, patients with minor injuries may attend specialist-run trauma clinics directly, without referral from primary care. These clinics generate approximately 170,000 additional acute outpatient contacts annually [37], which will be registered as specialist contacts with no referral and may obscure true prehospital referral pathways. However, our sensitivity analyses excluding Oslo University Hospital HF and Helse-Bergen HF, the two hospital trusts operating these services, demonstrated similar national trends across all outcomes. This suggests that the main findings were not driven solely by the organisational model or patient flow patterns in these two cities.

Most acute primary care referrals originated from office visits, although many referrals also followed remote contacts without physical meetings. The use of remote contacts for referrals increased during the study period. This pattern was evident for both telephone and online contacts with out-of-hours services, but only for online contacts with GP services. The apparent increase in telephone contacts in out-of-hours services may reflect greater use of the reimbursement codes rather than a true rise in contact rates, as a report from seven out-of-hours services in Norway shows relatively stable rates for telephone contacts [38]. Online consultations have been increasing since the COVID-19 pandemic [39,40], which aligns with our findings. This trend may have implications for our analyses. Nordberg et al. found that Norwegian GPs expressed concern that online consultations increased low-acute contacts, potentially impairing their ability to effectively identify serious conditions requiring specialist care [41]. The use of remote consultations for potentially lower acuity health issues may affect our analyses by implying a falsely high referral rate, as the chance of coincidental associations between primary care contacts and acute specialist contacts increases.

Our findings differ from a previous Norwegian study [6], which found that 28% of admissions occurring within 24 h of a primary care contact were GP-referred and 36% were out-of-hours physician-referred. This discrepancy likely reflects methodological differences: we included both acute outpatient specialist contacts and admissions, resulting in a higher overall number of specialist contacts. In addition, we defined a shorter 10-hour referral window, used a more narrow definition of primary care contacts, and included obstetric admissions—which decreased our proportion of primary care-referred contacts. Approximately 55,000 annual births in Norway follow standardized direct admission protocols without primary care referral, representing a substantial portion of acute contacts classified as ‘others’ in our study. During the study period, births declined slightly from 59,403 to 55,402 per year [42]. As these contacts bypass primary care, they represent a distinct pathway that is less directly relevant to a primary care perspective. However, their inclusion allows a more complete description of acute specialist activity without affecting estimates of primary care referrals. Nevertheless, their inclusion should be considered when interpreting the proportion of specialist contacts involving primary care.

Interestingly, while our data show a national decline in acute specialist contacts, a recent study documented an increase in emergency department visits at two Norwegian university hospitals during the same period [21]. This pattern of diverging trends—decreasing acute specialist contacts alongside increasing emergency department visits—has also been observed in Ontario, Canada [23]. Our definition of specialist contacts included all consultations involving specialist care, both in hospitals and outpatient settings, including acute services delivered by contract specialists operating on behalf of hospital trusts outside hospital facilities. However, the majority of acute specialist contacts occurred in hospitals, as contract specialists only accounted for a small proportion of the total (8.2%). Furthermore, our data did not distinguish whether specialist contacts (outpatient or admission) occurred through the emergency department or used other entry points. Although primary care may direct more patients to specialist care, our data cannot determine whether these referrals contribute to emergency department visits. These seemingly contradictory findings of decreased acute specialist contacts and increased emergency department visits may be explained by structural changes in the Norwegian healthcare system. Norway has centralised its healthcare system into fewer hospitals [5], with many hospitals now directing acute patients primarily through emergency departments. This centralisation consolidates smaller emergency departments into larger regional facilities serving multiple municipalities [5], increasing pressure on remaining facilities, particularly university hospitals [21].

The relationship between primary care access and hospital utilisation is complex. A systematic review from 2012 [8] found an association between limited primary care accessibility and avoidable hospitalisation, although this association was weaker in countries with gatekeeper systems. Morley et al. [22] identified in their 2018 systematic review that limited primary care access increased emergency department visits, based on findings from three studies [43–45]. However, evidence from the UK suggests that primary care access alone does not fully explain variations in emergency department use [46], indicating that other factors beyond accessibility influence referral patterns.

Norwegian studies have highlighted the importance of continuity in the GP-patient relationship for avoiding acute referrals [47]. This is particularly relevant given that Vinjerui et al. [48] demonstrated that disruptions in regular GP-patient relationships led to increased use of out-of-hours services, which our findings suggest may be important drivers of increased specialist referrals.

### 4.1. Strengths and limitations

The primary strength of this study is its comprehensive scope, encompassing the entire Norwegian population over a 10-year period. This provides robust national evidence of changing referral patterns in the acute care system.

The main limitation is reliance on registry data, which limits our analysis to what is recorded. Changes in coding practice, documentation routines, or local organisational differences may therefore be reflected in the data, even if the true number of clinical contacts remains unchanged.

Our data did not allow us to identify the specific referral pathway for acute specialist contacts without prior involvement of primary care (GP service or out-of-hours service) (categorised as ‘others’). This represents an important patient group that should be addressed in future research. Assuming that primary care contacts occurring within 10 h represent referrals may have introduced some false linkages. However, the shorter time window reduced this risk compared with previous studies using longer windows. Conversely, acute referrals made in the evening for specialist assessment in the following morning would not be captured in the data and therefore excluded as a referral (categorised as ‘others’). Scheduling semi-acute specialist assessments for the following morning is a common arrangement for non-life-threatening conditions. We also included delayed claims submitted up to 8 h after the specialist contact to account for reporting delays. Although some false-positive and false-negative linkages may have occurred, we believe the overall trends are unlikely to be materially affected. These registries primarily serve administrative and reimbursement purposes, which likely promotes a high degree of contact reporting. However, we selectively included KUHR reimbursement codes based on their definition that could plausibly lead to referrals, while excluding codes for routine services such as blood tests or prescriptions. Alternative code selections may have produced different proportions of linked specialist-primary care contacts. Nevertheless, the consistency of findings across analyses supports the robustness of the observed trends. Finally, our data did not distinguish between emergency department visits and other hospital contacts, which would have been valuable in addressing the observed discrepancy between emergency department visits and acute hospital contacts.

## 5. Conclusions

Based on national registry data, the incidence rate of acute specialist contacts following a primary care contact has increased over the past decade in Norway. In the recorded data, the rise appears to be mostly driven by prior contacts with out-of-hours services, partly reflecting a temporary increase during the COVID-19 pandemic when these services were expanded. During the same period, there has been a decrease in acute specialist contacts, both in absolute numbers and population-adjusted rates, indicating that primary care has become a more frequent pathway to acute specialist care. The trends were consistent across all demographic variables examined and likely reflect structural shifts in the organisation of healthcare or changes in coding and registration practices, rather than changes in the population itself.

As the analysis relies on recorded registry activity, changes in coding and documentation practice during the study period may influence the observed trends. In addition, future research should explore patterns of nursing home referrals and emergency medical service transports to hospitals, while distinguishing whether the ‘specialist contact’ occurred at a hospital ward, a specialist outpatient clinic, or the emergency department.

## Supplementary Material

Supplementary file 1.pdf

Supplementary file 2.pdf

Supplementary file 3.pdf

Supplementary files (1).xlsx

## Data Availability

The data that support the findings of this study are available from The Norwegian Directorate of Health (www.helsedirektoratet.no) and Statistics Norway (www.ssb.no) but restrictions apply to the availability of these data, which were used under license for the current study, and so are not publicly available. Data are, however, available from the authors upon reasonable request and with permission of The Norwegian Directorate of Health, Statistics Norway, the Regional Ethical Committee, and the Norwegian Data Protection Authority.
