# Supplementary material for: Trends in acute specialist contacts following a primary care contact 2012–21—a registry-based study
Source: Scand J Prim Health Care. 2026 Jun 2;44(1):2677785. doi: 10.1080/02813432.2026.2677785 (PMC13231808; doi:10.1080/02813432.2026.2677785)
Supplement: Supplementary file 1.pdf [file IPRI_A_2677785_SM6259.pdf]

Supplementary file 1. Contact type based on reimbursement codes by The Norwegian Health Economics Administration (Helfo)

The reimbursement codes were first separated by type of service (general practitioner service or out-of-hour service), based on the variable “praksis refusjonsgrunnlag”.

Contact type	Meaning	Reimbursement codes
Office	Physician’s appointment where the patient met the physician at an office	2ad, 2ak, 2nk, 2fk, 1ad, 1ak
Telephone	Physician’s appointment where the consultation took place by telephone	1g, 1bk,1bd
Home visit	Physician’s appointment where the consultation took place at the patient’s home	11ak, 11ad
Online contact*	Physician’s appointment where the consultation took place electronical	2aek, 2ae

*In use from 2013
