# Supplementary material for: Trends in acute specialist contacts following a primary care contact 2012–21—a registry-based study
Source: Scand J Prim Health Care. 2026 Jun 2;44(1):2677785. doi: 10.1080/02813432.2026.2677785 (PMC13231808; doi:10.1080/02813432.2026.2677785)
Supplement: Supplementary file 2.pdf [file IPRI_A_2677785_SM6258.pdf]

Supplementary file 2 - Estimated incidence rates (IRs) of acute specialist contacts in total and following a primary care contact[1], per 1,000 person-years

Acute specialist contacts
IR (95% CI)

	Total		Outpatient contacts		Admissions	
**Year**	**National**	**Excluding OUS* HF and Helse-Bergen HF**	**National**	**Excluding OUS* HF and Helse-Bergen HF**	**National**	**Excluding OUS* HF and Helse-Bergen HF**
2012	311.3 (310.1–312.5)	218.0 (217.2–218.9)	193.5 (192.4–194.6)	112.8 (112.2–113.5)	119.7 (119.3–120.2)	106.4 (106.0–106.9)
2013	312.8 (311.6–314.0)	216.3 (215.4–217.1)	189.8 (188.7–190.9)	106.5 (105.9–107.2)	123.3 (122.8–123.8)	110.0 (109.6–110.5)
2014	287.9 (286.8–289.0)	220.7 (219.9–221.5)	160.3 (159.4–161.2)	107.1 (106.5–107.7)	126.8 (126.2–127.3)	113.3 (112.8–113.8)
2015	252.6 (251.6–253.5)	183.3 (182.6–183.9)	147.9 (147.1–148.6)	92.7 (92.3–93.1)	103.1 (102.7–103.6)	89.6 (89.2–90.0)
2016	226.8 (226.1–227.6)	175.2 (174.6–175.8)	119.7 (119.2–120.1)	82.1 (81.8–82.5)	106.3 (105.8–106.8)	92.4 (91.9–92.8)
2017	223.0 (222.3–223.8)	169.7 (169.0–170.3)	112.2 (111.8–112.7)	73.9 (73.5–74.2)	110.5 (110.0–111.1)	95.8 (95.3–96.3)
2018	227.6 (226.9–228.4)	175.2 (174.5–175.9)	113.0 (112.5–113.4)	76.1 (75.7–76.4)	114.8 (114.2–115.4)	99.3 (98.7–99.8)
2019	234.5 (233.7–235.3)	181.7 (181.0–182.4)	116.1 (115.7–116.6)	78.9 (78.6–79.3)	118.8 (118.2–119.5)	102.9 (102.3–103.5)
2020	210.6 (209.9–211.4)	166.4 (165.8–167.1)	99.5 (99.1–99.9)	69.3 (69.0–69.7)	112.9 (112.2–113.5)	98.1 (97.5–98.7)
2021	227.6 (226.8–228.4)	179.8 (179.1–180.4)	108.7 (108.3–109.2)	75.7 (75.3–76.0)	120.7 (120.0–121.3)	105.1 (104.4–105.7)

Acute specialist contacts following a primary care contact[2]
IR (95% CI)

	Total		Outpatient contacts		Admissions	
**Year**	**National**	**Excluding Helse-Bergen HF and OUS HF**	**National**	**Excluding Helse-Bergen HF and OUS HF**	**National**	**Excluding Helse-Bergen HF and OUS HF**
2012	91.8 (91.5–92.1)	80.4 (80.1–80.7)	38.4 (38.2–38.6)	33.5 (33.3–33.7)	52.6 (52.3–52.8)	46.9 (46.7–47.2)
2013	95.7 (95.3–96.0)	83.6 (83.3–83.9)	39.9 (39.7–40.1)	34.7 (34.6–34.9)	54.6 (54.3–54.8)	49.0 (48.7–49.2)
2014	96.4 (96.0–96.7)	85.5 (85.2–85.9)	39.9 (39.7–40.1)	35.9 (35.7–36.0)	55.4 (55.1–55.6)	49.9 (49.6–50.1)
2015	98.2 (97.8–98.6)	86.6 (86.2–86.9)	41.4 (41.2–41.6)	36.6 (36.5–36.8)	55.9 (55.6–56.2)	50.2 (49.9–50.4)
2016	98.9 (98.5–99.3)	87.4 (87.1–87.7)	41.1 (40.9–41.3)	36.6 (36.4–36.7)	57.1 (56.8–57.4)	51.2 (50.9–51.4)
2017	102.1 (101.7–102.5)	90.4 (90.0–90.7)	43.0 (42.8–43.1)	38.2 (38.1–38.4)	58.6 (58.2–58.9)	52.5 (52.2–52.8)
2018	106.4 (106.0–106.8)	93.0 (92.6–93.4)	46.0 (45.8–46.2)	40.0 (39.9–40.2)	59.7 (59.4–60.1)	53.3 (53.0–53.7)
2019	109.9 (109.4–110.3)	95.6 (95.2–96.0)	48.4 (48.2–48.6)	41.8 (41.6–42.0)	60.7 (60.3–61.0)	54.1 (53.8–54.5)

2020	101.0 (100.5–101.4)	87.1 (86.6–87.4)	43.5 (43.3–43.7)	36.9 (36.7–37.1)	56.7 (56.3–57.0)	50.6 (50.2–50.9)
2021	109.3 (108.8–109.7)	93.8 (93.4–94.3)	48.2 (48.0–48.4)	40.7 (40.5–40.9)	60.1 (59.7–60.5)	53.5 (53.1–53.9)

[1] The primary care contact was registered within 10 hours prior to the acute hospital contact

[2] General practitioner service or out-of-hours service

* Oslo University Hospital
