# Supplementary material for: Trends in acute specialist contacts following a primary care contact 2012–21—a registry-based study
Source: Scand J Prim Health Care. 2026 Jun 2;44(1):2677785. doi: 10.1080/02813432.2026.2677785 (PMC13231808; doi:10.1080/02813432.2026.2677785)
Supplement: Supplementary file 3.pdf [file IPRI_A_2677785_SM6257.pdf]

Supplementary file 3 - Estimated incidence rates (IRs) of acute specialist contacts, by type of prior primary care contact and no prior primary care contact[1], per 1,000 person-years

	GP service[2], IR (95% CI)		Out-of-hours service, IR (95% CI)		No prior contact (others), IR (95% CI)	
Year	National	Excluding OUS* HF and Helse-Bergen HF	National	Excluding OUS* HF and Helse-Bergen HF	National	Excluding OUS* HF and Helse-Bergen HF
2012	45.1 (44.9–45.3)	39.7 (39.5–39.9)	46.6 (46.4–46.8)	41.3 (41.1–41.5)	222.1 (221.0–223.2)	139.2 (138.5–140.0)
2013	46.4 (46.2–46.6)	40.9 (40.7–41.1)	48.6 (48.3–48.8)	43.0 (42.8–43.2)	218.7 (217.6–219.9)	132.9 (132.2–133.6)
2014	46.5 (46.3–46.7)	41.8 (41.6–42.0)	48.7 (48.5–49.0)	43.6 (43.3–43.8)	191.7 (190.7–192.6)	134.1 (133.4–134.8)
2015	46.4 (46.2–46.6)	41.4 (41.2–41.6)	50.0 (49.8–50.3)	44.5 (44.3–44.7)	153.2 (152.4–154.0)	94.5 (94.0–95.0)
2016	46.1 (45.9–46.3)	41.5 (41.3–41.7)	50.6 (50.4–50.9)	44.9 (44.6–45.1)	125.4 (124.9–126.0)	84.7 (84.3–85.2)
2017	47.0 (46.8–47.3)	42.4 (42.2–42.7)	52.5 (52.2–52.7)	46.4 (46.2–46.7)	117.5 (117.0–118.0)	75.4 (75.0–75.8)
2018	47.6 (47.4–47.8)	43.1 (42.8–43.3)	55.7 (55.4–56.0)	48.1 (47.8–48.3)	117.0 (116.5–117.6)	77.4 (77.0–77.8)
2019	48.0 (47.9–48.3)	43.6 (43.3–44.0)	59.3 (59.0–59.6)	49.8 (49.5–50.0)	119.6 (119.1–120.1)	80.5 (80.0–80.9)
2020	43.4 (43.2–43.7)	39.6 (39.3–39.8)	53.7 (53.4–54.0)	45.1 (44.8–45.3)	104.0 (104.4–105.4)	73.8 (73.4–74.2)
2021	47.0 (46.8–47.3)	42.9 (42.6–43.1)	58.1 (57.8–58.4)	48.3 (48.0–48.6)	112.9 (112.4–113.5)	79.6 (79.2–80.0)

[1] The primary care contact was registered within 10 hours prior to the acute hospital contact

[2] General practitioner

* Oslo University Hospital
